# Symplasmata are a clonal, conditional, and reversible type of bacterial multicellularity

**DOI:** 10.1038/srep31914

**Published:** 2016-08-18

**Authors:** Robin Tecon, Johan H. J. Leveau

**Affiliations:** 1 Department of Plant Pathology, University of California, One Shields Ave, Davis, CA 95616, USA; 2 Department of Microbial Ecology, Netherlands Institute of Ecology (NIOO-KNAW), Droevendaalsesteeg 10, 6708 PB Wageningen, the Netherlands

## Abstract

Microorganisms are capable of remarkable social behaviours, such as forming transient multicellular assemblages with properties and adaptive abilities exceeding those of individual cells. Here, we report on the formation and structure of genets known as symplasmata produced by *Pantoea eucalypti* bacteria. Each symplasmatum develops clonally and stochastically from a single bacterium into a membrane-delimited, capsule-embedded cluster of progeny cells and with a frequency that depends on temperature, pH, and nutrient availability. Transposon mutagenesis identified several gene products required for symplasmata formation, including master regulator LrhA, replication inhibitor CspD, polysaccharide transporter RfbX3, and autoinducer synthase PhzI. We also show that bacteria inside symplasmata are shaped irregularly with punctuated cell-to-cell contacts, metabolically responsive to environmental stimuli, dispersal-ready, and transcriptionally reprogrammed to anticipate multiple alternative futures in terms of carbon source availability. The structured and conditionable nature of symplasmata offers exciting prospects towards a mechanistic understanding of multicellular behaviours and their ecological significance.

Many examples of multicellularity exist among bacteria^1^,^2^,^3^, and typically these represent specialized or obligate lifestyles. In this study, we focus on a facultative and transient form of multicellularity observed in bacteria belonging to the genus *Pantoea*. This genus (Family: Enterobacteriaceae) comprises bacteria that can be isolated from a wide variety of environments (soil, water, hospitals) and hosts (insects, animals, plants)^4^,^5^. The type species of the genus is *Pantoea agglomerans* (*P. agglomerans*)^6^. The epithet ‘*agglomerans*’ (‘forming into a ball’) dates back to Beijerinck’s original description of the species (as *Bacillus agglomerans*) in 1888^7^ and likely refers to the ability of *P. agglomerans* strains to form multicellular clusters known as symplasmata, also described as zoogloea, sausage forms, caterpillar formations (“raupchenartige Bildungen”), and “Bakterien-verbanden”^8^,^9^,^10^,^11^,^12^. The taxonomic history of *Pantoea* isolates is complex^4^, and in recent years several new *Pantoea* species have been proposed^5^. As a consequence, many strains previously known as *P. agglomerans* (and capable of forming symplasmata) are now referred to by newer species names, for example *P. eucalypti* 299R (formerly known as *P. agglomerans* 299R and before that *Erwinia herbicola* 299R^13^,^14^,^15^,^16^,^17^,^18^), *P. vagans* C9-1^19^ (once known as *P. agglomerans* C9-1^20^) and *P. anthophila* Sc1 (previously known as *P. agglomerans* Sc1).

The ecological role and evolutionary beginnings of symplasmata formation in *Pantoea* bacteria remain unclear. There have been reports that these structures may confer tolerance to stresses such as acids, heavy metals and UV^21^. For plant-colonizing *P. agglomerans* strains, symplasmata have been directly observed on the surfaces of roots and leaves, as well as within plant tissues^21^,^22^,^23^,^24^,^25^,^26^,^27^,^28^. Despite the fact that symplasmata have been known for more than a century, our understanding of the genes that underlie their formation is scarce, which limits our ability to formulate and test hypotheses on the ecology and evolution of symplasmata formation. Here, we present the results from a study on symplasmata formation in *P. eucalypti* 299R (*Pe*299R), which is a model strain for the study of bacterial colonization of the phyllosphere, i.e. plant leaf surfaces^14^,^29^,^30^. We offer an in-depth analysis of the formation, structure, and genetics of *Pe*299R symplasmata and discuss in more general terms the formation of symplasmata as a survival strategy in changing and unpredictable environments.

## Results and Discussion

**Figure 1:**
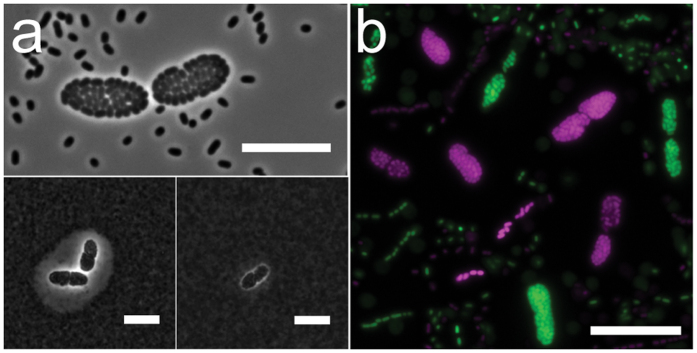
Symplasmata formation by *Pantoea eucalypti* 299R. (**a**) Top: phase-contrast image showing a pair of symplasmata amidst single cells. Bottom: counter-staining with indian ink reveals a capsule surrounding the cells clusters (left) which is gone after one hour incubation with sodium metaperiodate (right). Bars 10 μm. (**b**) Merged fluorescent image of a mixed culture of *P. eucalypti* cells expressing either GFP (pseudo-colored green) or DsRed (pseudo-colored pink). Each symplasmatum contains cells of one color only. Bar 20 μm. The bacteria shown in panels a and b were taken from liquid cultures growing on M9 plus glucose.

**Figure 2:**
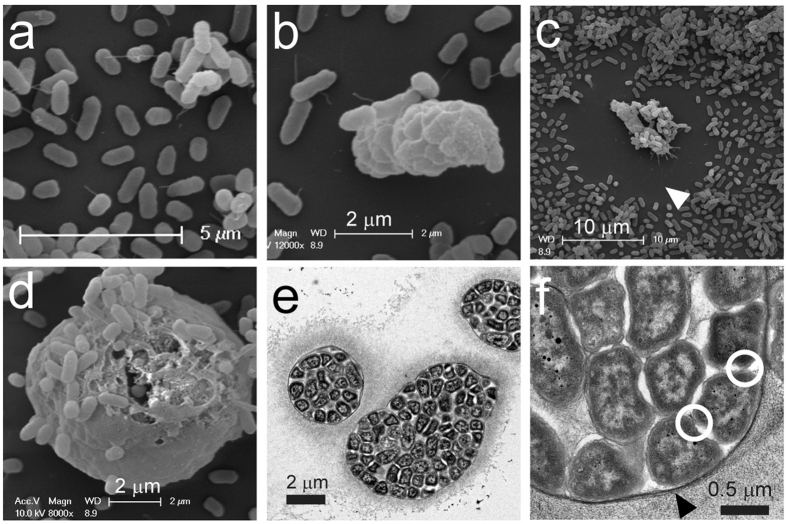
Scanning (**a**–**d**) and transmission (**e**–**f**) electron micrographs of single cells (**a**) and symplasmata (**b**–**f**) formed by *Pantoea eucalypti*. (**b,c**) Symplasmata at an early developmental stage. White arrowhead indicates an area of exclusion where a capsule would be expected but was removed during preparation for electron microscopy. (**d**) Close-up of a capsule-stripped mature symplasmatum revealing a distinct membrane-like layer keeping together the cells inside. (**e**) Cross-sectional view of symplasmata with packed cells and capsular matrix. (**f**) Close-up of a symplasmatum cross-section. Arrowhead indicates the membrane-like layer holding the cells together, while white circles point to two of the cell-to-cell contact points. The bacteria shown in panels a–f were taken from liquid cultures growing on M9 plus glucose.

In our laboratory, we have observed symplasmata formation by *Pe*299R in liquid cultures, on agar plates, and on the leaf surfaces of green bean plants. Typically, each symplasmatum contained several to hundreds of bacterial cells inside a shared capsule that is up to 20 micrometers thick as visualized by ink counterstaining or transmission electron microscopy (Figs 1 and 2). Capsule material was composed of a glycoconjugate containing sugars (most notably rhamnose, glucose and galactose), uronic acids, glycerol, and inositol (Supplementary Table S1, online). Incubation with metaperiodate, a chemical that digests polysaccharides^31^, removed the capsule, but did not release cells from the clusters (Fig. 1a). Capsular material was also removed during preparation for scanning electron microscopy, leaving a characteristic ‘void space’ where capsules would be expected (Fig. 2c) and showing the symplasmata as spheroids or allantoids with a membrane-like smooth surface (Fig. 2b–d) that proved resistant to treatment with proteinase K (not shown) or staining with a lipophilic fluorescent dye (Supplementary Fig. S1). Occasionally, this membrane ruptured and curled, offering a view of the bacteria inside the symplasmatum (Fig. 2d). Transmission electron microscopy showed the membranes as electron-dense and oftentimes stacked layers (Fig. 2e,f). Bacteria within each symplasmatum displayed an irregular shape and punctuated cell-to-cell contact with neighboring bacteria (Fig. 2f).

**Figure 3:**
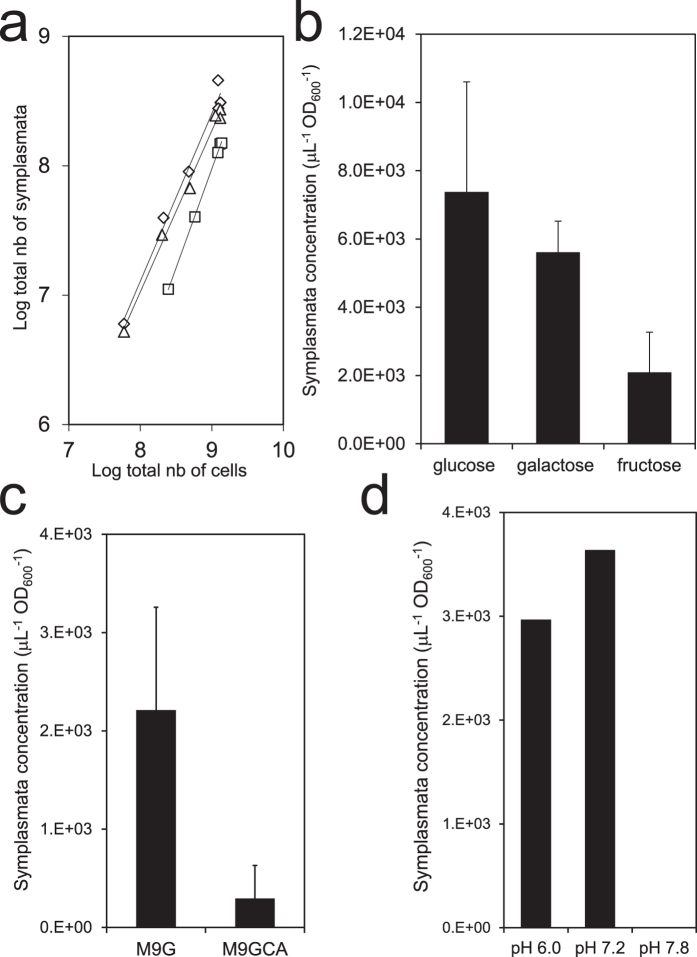
Concentration of *Pantoea eucalypti* symplasmata in liquid cultures. (**a**) Total number of symplasmata against total number of bacterial cells in minimal medium with 0.4% glucose. Diamonds, squares and triangles represent three biological replicate experiments. (**b**) Symplasmata concentration in mid-log phase cultures with different carbon sources (0.4%). Error bar is one standard deviation calculated from triplicate measurements. (**c**) Symplasmata concentration after 24 hours of incubation in minimal medium with 0.4% glucose in the presence (M9GCA) or absence (M9G) of 0.2% casamino acids. Data shown represent the mean of independent biological replicates in flasks (M9G: 8; M9GCA: 5). Error bars represent one standard deviation. (**d**) Symplasmata concentration after 24 hours of incubation in M9G set with various pH (results from one experiment are shown).

**Figure 4:**
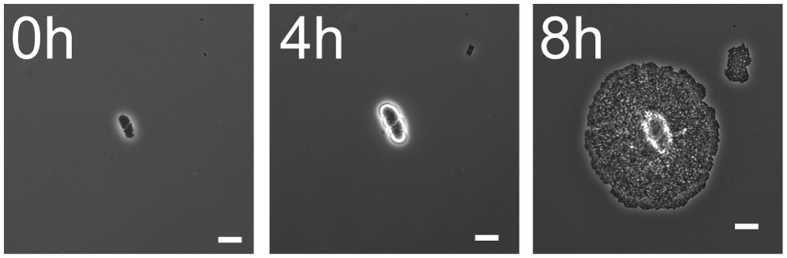
Reversion from symplasmatal to solitary growth. Micrographs show a one week-old symplasmatum from liquid culture inoculated onto the surface of a M9GCA 1% agarose gel and incubated at room temperature. Between 4 and 8 hours, cells burst from the symplasmatum, and resumed growth to form a microcolony. Bar is 10 μm. Also see Supplementary Movie S1.

In liquid media, symplasmata formation varied with culture conditions. During exponential growth (*μ* = 0.64 ± 0.02 hr^−1^) in minimal medium with glucose, at pH 7.2, 30 °C and with shaking, the total number of symplasmata increased exponentially (Fig. 3a). The ratio of symplasmata to solitary cells was relatively constant at about 1 to 200, with symplasmata appearing at a rate of 0.003 ± 0.001 hr^−1^. Similar rates were observed with galactose or fructose as sole carbon source, although fructose led to slightly lower symplasmata concentrations (Fig. 3b). Addition of casamino acids (hydrolysate of casein) to the medium significantly reduced symplasmata concentration (Fig. 3c). Lowering the pH to 6.0 had little effect. However, at pH 7.8 symplasmata formation was almost totally abolished (Fig. 3d). We did not observe symplasmata in cultures grown in rich medium (Lysogeny Broth) or at 37 °C (data not shown). These results confirm that symplasmata formation is a conditional phenotype influenced by nutrient and physicochemical factors. Cells inside symplasmata remained responsive to stimulation with chemical signals in the medium (Supplementary Fig. S2) and they were able to replicate upon release from the cluster (Fig. 4). Bacteria typically left symplasmata in rapid bursts and immediately started to multiply (Fig. 4, Supplementary Movie S1), demonstrating the reversible nature of symplasmata and the return of cells to a free-living lifestyle.

**Figure 5:**
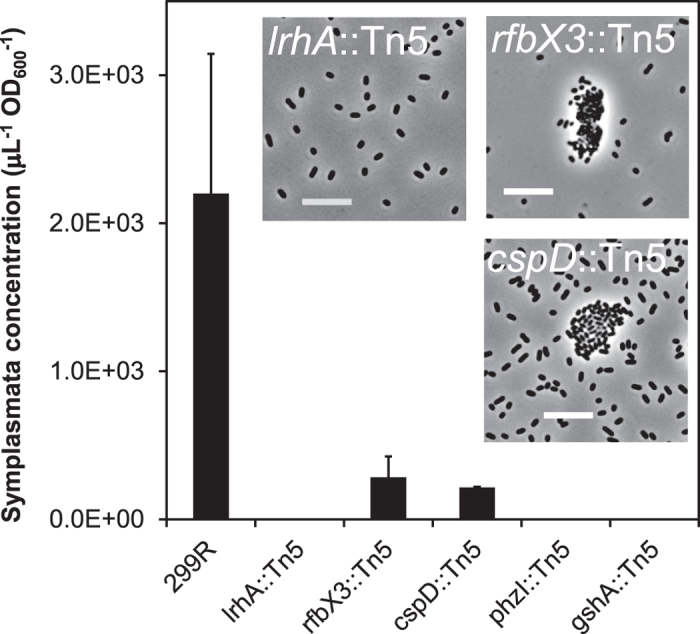
Symplasmata formation by wildtype and Tn*5* insertion mutants of *Pantoea eucalypti*. The concentration of symplasmata was measured after 24 hours of incubation in liquid medium. Error bars represent one standard deviation calculated from 11 replicates (wild-type *P. eucalypti* 299R), duplicates (*cspD*::Tn*5*), or triplicates (all other strains). Inserted micrographs show the phenotype of mutants *lrhA*::Tn*5*, *rfbX3*::Tn*5*, and *cspD*::Tn*5* (note: *phzI*::Tn*5* and *gshA*::Tn*5* are not shown as they looked identical to *lrhA*::Tn5). Bars are 10 μm.

The ability to form symplasmata was abolished, reduced, or otherwise altered in several transposon insertion mutants of *Pe*299R (Fig. 5, Supplementary Table S2). From a library of 4,981 mutants, we identified three independent transposon insertions in the *lrhA* gene (NCBI gene locus_tag: F385_RS17575) which were linked to complete loss of clustering phenotype, but could be restored by full gene complementation (Supplementary Fig. S3). The *lrhA* gene encodes the LysR-type regulatory protein LrhA. This protein is conserved among Enterobacteriaceae as a transcriptional repressor of *flhDC*, which codes for the master regulator of flagellar, motility and chemotaxis genes^32^. Consistent with this, the *lrhA* mutants of *Pe*299R exhibited increased motility on soft agar (Supplementary Fig. S4) and elevated expression of genes belonging to the *flhDC* regulon, including *flh*, *flg*, *che*, *mot*, *fli, tsr*, and *aer* genes (see Supplementary Table S3). In *E. coli*, LrhA also stimulates the rate of phase variation to a pilus-mediated adhesive phenotype^33^. In the *Pe*299R *lrhA* mutant, the fimbriae assembly genes *pilB* and *pilC* were indeed downregulated compared to wildtype (Supplementary Table S3). Phase variation is a mechanism that allows bacteria to introduce phenotypic heterogeneity into a population of genotypically identical cells^34^. Such a mechanism may explain the co-existence of solitary cells and symplasmata in culture. It also has been reported that LrhA exerts quorum sensing-dependent control over capsule production and motility in the plant pathogen *Pantoea stewartii* subsp. *stewartii*^35^,^36^. Other genes that were differentially expressed in the *lrhA* mutant (compared to wildtype) were *yfiA* (coding for a ribosome hibernation protein), *spoVR* (sporulation family protein), *pflB* (pyruvate formate lyase), *focA* (formate efflux transporter), *cydAB* (cytochrome d), and *yqhE* (also known as *dkgA*) (Supplementary Table S3). Expression of three of these genes (*pflB*, *focA* and *cydB*) has been reported to increase under anaerobiosis^37^,^38^ or micro-aerobiosis^39^, which aligns with the notion^24^ that cells inside symplasmata experience conditions of reduced oxygen levels.

Transposon insertions in the *cspD* gene (locus_tag: F385_RS03285) or in the *rfbX3* gene (locus_tag: F385_RS21230) reduced or altered but did not abolish symplasmata formation (Fig. 5, Supplementary Table S2). In *E. coli*, CspD is a stationary-phase, replication inhibition protein that plays a role in the formation of bacterial persisters^40^, i.e. cells that go quiescent to survive stresses but can regrow under favorable conditions^41^. The *rfbX3* gene codes for a predicted Wzx-like inner-membrane transporter of polysaccharidal O-antigens. Functional restoration of the *rfbX3* mutant required both *rfbX3* and its downstream gene which is predicted to code for a glycosyltransferase (Supplementary Fig. S3). In other mutants, Tn*5* insertion sites could be mapped to *phzI* (coding for an *N*-acyl-homoserine lactone (AHL) synthase; locus_tag: F385_RS04930), *gshA* (gamma-glutamylcysteine synthase, involved in glutathione biosynthesis; locus_tag: F385_RS02625), *yhdP* (sugar transporter; locus_tag: F385_RS20315), and *rodA* (cell-shape determining protein; locus_tag: F385_RS16900). The apparent requirement of *phzI* for symplasmata formation in *P. eucalypti* suggests a role for quorum sensing. AHLs have been previously linked to production of capsular exopolysaccharides in closely related species such as *Pantoea ananatis*^42^ and *Pantoea stewartii*^43^, and have recently been shown to play a role in symplasmata formation by *P. agglomerans* YS19^27^. Complementation of the *phzI* mutant with a full-length *phzI* gene and its native promoter restored symplasmata formation to wildtype levels (Supplementary Fig. S3). We were unable to restore symplasmata formation in the *gshA* and *cspD* mutants by full gene complementation (data not shown).

**Figure 6:**
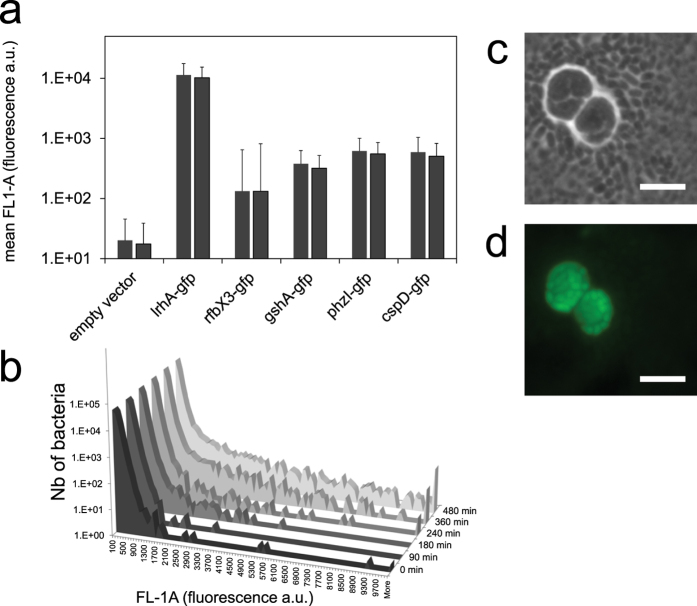
Expression of symplasmata-associated genes in *Pantoea eucalypti* as measured by GFP reporter fusions. (**a,b**) Intensity of GFP fluorescence measured by flow cytometry in individual *Pe*299R bacteria carrying a reporter plasmid. (**a**) Histograms show the mean GFP fluorescence intensity (FL1-A) in individual bacteria after 8 hours of growth in M9G liquid cultures, expressed as arbitrary units and calculated from a sample of 10,000 cells; bars show results from two independent experiments; error bar indicates 1 SD. (**b**) Count histograms of *Pe*299R bacteria carrying the pRfbX3-gfp plasmid; we show the distribution of 100,000 cells after 0, 1.5, 4, 6 and 8 hours of incubation. (**c,d**) Micrographs of *Pe*299R (pRfbX3-*gfp*) after 21 hours of growth on the surface of M9G agarose. A symplasmatum surrounded by single cells is shown. Phase contrast (**c**) and GFP fluorescence with pseudo-color green (**d**) are shown. Bar is 5 μm.

Using a *gfp* reporter gene, we constructed transcriptional fusions to study the expression of *lrhA*, *rfbX3*, *cspD*, *phzI* and *gshA* in *Pe*299R (Fig. 6). Each reporter fusion was carried on a separate plasmid (pLrhA-gfp, pRfbX3-gfp, pCspD-gfp, pPhzI-gfp and pGshA-gfp, see Supplementary Table S6), and GFP fluorescence intensity was assessed in individual cells by fluorescence microscopy and flow cytometry. All reporter fusions showed fluorescence intensities above the background signal of the control plasmid (empty vector), with the lrhA-gfp fusion producing the highest intensity level (Fig. 6a). This supports the view that in *Pe*299R these symplasmata-associated genes are constitutively expressed during growth in minimal medium with glucose, albeit at levels that vary greatly. Interestingly, the variation in GFP expression among individual bacteria was highest with the rfbX3-gfp fusion (as shown by the error bars in Fig. 6a), and coincided with the appearance over time of a subpopulation of cells showing elevated GFP expression (Fig. 6b). Using fluorescence microscopy, we showed that GFP expression from the rfbX3-gfp fusion was induced only in cells within *Pe299R* symplasmata, and not in solitary cells (Fig. 6c,d), which is evidence that *rfbX3* is specifically expressed in symplasmata. It also appears that activation of *rfbX3* is an important event in the formation of symplasmata and occurs early on in this process (Supplementary Fig. S5). RNA-Seq analysis showed that *rfbX3* was downregulated (p-value = 0.024) in the *lrhA* mutant (which cannot form symplasmata) compared to wildtype. Expression of *rfbX3* was observed frequently in stringed groups of 2, 4, 8, or 16 cells, which suggests synchronized cell division during the very early stages of symplasmata formation (Supplementary Fig. S5). Starting at the 8-cell stage, this synchrony appeared to break down and cell packing started to look more irregular, as it does in mature symplasmata. This notion of symplasmata as ‘genets’, i.e. groups of genetically identical individuals, growing together and originating from a single ancestor, is consistent with the observation that in mixed cultures of red and green fluorescent *Pe*299R, individual clusters never consisted of differently colored cells (Fig. 1b), confirming the hypothesis of Achouak *et al*.^22^ that each symplasmatum is clonal, i.e. derived from a single bacterial progenitor.

The transcriptional profile of cells within symplasmata was markedly different from that of solitary cells, as revealed by reporter gene fusions (Fig. 6) and RNA-Seq analysis (Supplementary Fig. S6, Supplementary Table S4). Cells in symplasmata showed 343 differentially expressed genes (p-value < 0.05) compared to single cells, which corresponded to 8% of *Pe*299R genes, of which half were upregulated and half were downregulated. Many of the genes that were upregulated (≥5-fold, p-value < 0.05) code for structural or regulatory proteins involved in the uptake or degradation of carbon substrates, including galactose (*mglB*, *mglA*, *mglD*), galactofuranose/pyranose (*ytfQ*), rhamnose (*rhaT*, *rhaS*), arabinose (*araC*), ribose (*rbsK*), maltose (*malE*), glucuronate/galactonate (*uxuA*), glycerol 3-phosphate (*glpT*), glycerol (*glpF*), inositol (*iolD*, *iolI*), and fatty acids (*fadL*, *fadI*, *fadD*). Interestingly, many of these compounds were also present in the symplasmatal capsule material (Supplementary Table S1), and most of the cognate genes are predicted to be regulated by the cAMP receptor protein (CRP)^44^,^45^. In addition to genes involved in carbon uptake and metabolism, upregulated genes in symplasmata included *cspD* and other stationary phase-inducible, CRP-dependent^46^ genes (e.g. *csiE*, *cstA*), as well as *ompR1*, which encodes a putative response regulator of unknown function that has 97% amino acid similarity with OmpR1 in *Pantoea vagans* C9-1. We did not observe significant differences in the expression of other symplasmata-associated genes, including *rfbX3*. This could indicate that *rfbX3* expression is transient and mostly required during the early stages of symplasmata formation, as previously noted. Finally, the chemotaxis genes *cheRBYZ* were slightly downregulated in symplasmata compared to single cells, whereas they were highly upregulated in the *lrhA* mutant compared to wildtype (Supplementary Table S3 and S4).

While symplasmata formation by *Pe*299R on leaf surfaces was observed in a lab-controlled environment (not shown), we do not know whether this phenotype is expressed on plant leaves in nature. However, symplasmata formation was common among *Pantoea* strains (*P. agglomerans* and *P. anthophila*) isolated freshly from the leaf surfaces of field-grown and harvest-ready lettuce (Supplementary Table S5), so we can rule out that symplasmata formation is a trait resulting from the domestication of model bacteria like *Pe*299R. Reports of symplasmata formation in natural environments by other authors^22^,^23^,^24^ also support this view. Moreover, we observed symplasmata in lab cultures of the fireblight-control strain *P. vagans* (formerly *agglomerans*^20^) C9-1^19^,^47^.

The data we present here are consistent with a model of symplasmata formation in which a small subpopulation of solitary cells enters a developmental program that involves the ‘staying-together’^48^ of each cell and its subsequent offspring. The precise mechanism that underlies this transition to multicellularity cannot presently be inferred, but it is responsive to environmental clues (e.g., pH, nutrients) and possibly entails LrhA-dependent, pilus-mediated aggregation^49^. Part of the symplasmatal developmental program in *P. eucalypti* involves activation of *rfbX3* and its downstream gene to generate an extracellular capsule that covers the cells within each symplasmatum. The glycosyl residues found in the extracellular polymeric substances produced by *Pe*299R share similarities with the chemical composition of stewartan and amylovoran, two exopolysaccharides produced by *Pantoea* and *Erwinia* species^50^. Inside the capsule, *Pe*299R cells continue to multiply and eventually are starved for glucose and primed for utilization of other carbon sources, including those that are stored in the capsule. In *E. coli*, glucose-limiting conditions are known to induce the expression of multiple genes for the uptake of alternative carbon sources, offering the cells a fitness advantage upon presentation of those sources^51^. Symplasmata formation might thus represent a possible bet-hedging strategy^52^ in which a subset of the population undergoes a multicellular-staged transcriptional reprogramming that prepares cells for multiple alternative futures. Such a strategy is likely to be advantageous to *P. eucalypti* in its native habitats, including the plant leaf surface, where nutritional conditions fluctuate both spatially and temporally^53^. Achouak *et al*. showed that *P. agglomerans* NO30 forms symplasmata upon contact with the roots of its host plant (rice), but not wheat (non-host)^22^,^23^, which suggests that symplasmata formation can be modulated by plant interactions. It was suggested that the symplasmatal capsule improves attachment of *P. agglomerans* to rice roots^22^. We were able to corroborate this for *P. eucalypti* by showing that symplasmata were harder to remove from surfaces than solitary cells (Supplementary Fig. S7). Recently, it was shown that cells inside of symplasmata were more resistant to stress than solitary cells^21^. Taken together, these observations by us and those of others suggest that symplasmata may play different, but not mutually exclusive roles in the ecology of *P. eucalypti* and *P. agglomerans* in their native habitats.

We have described in this study the specific characteristics of symplasmata: clonal, conditional, reversible, but also stochastic and probably adaptive. In our view, this makes symplasmata formation an attractive phenotype to advance the study of bacterial multicellularity. The highly structured nature of symplasmata (i.e. cells on the inside, capsular material on the outside, separated by a membrane-like entity) fits the definition of patterned multicellularity^2^, with the membrane providing a clear boundary to what is ‘self’ and what is not^54^. The organization of cells within each symplasmatum also hints at a basic level of coordination. The possible involvement of bacterial cell-to-cell junctions in this coordination will require further scrutiny, particularly in light of the predicted advantages of resource exchange between cells inside of a cluster^55^. Our careful characterization of *P. eucalypti* symplasmata offers new insights into a century-old observation, and paves the way for exciting new studies on bacterial multicellularity and its ecological role in environmental habitats.

## Methods

### Bacterial strains, culture conditions, and plasmid constructs

Bacterial strains and plasmids used in this study are listed in Supplementary Table S6. Rifampicin (Rif)-resistant *Pantoea eucalypti* strain 299R^16^ (abbreviated here as *Pe*299R) and its kanamycin (Km)-resistant plasmid- or Tn*5*-derivatives (see below) were routinely grown in Lysogeny Broth (LB) with shaking at 275 rpm and 30 °C, or on LB agar plates at 28 °C. Final concentrations of Rif and Km were 20 and 50 μg per ml, respectively. Defined medium was M9^56^ supplemented with 0.4% glucose, without (M9G) or with (M9GCA) 0.2% casamino acids (casein hydrolysate) (Fischer Bioreagents, Fair Lawn, USA). Strains *Pe*299R (pFRU48) and *Pe*299R (pFRU97) constitutively express green and red fluorescent protein, respectively^29^, and were used to demonstrate the clonal nature of symplasmata as described in the Supplementary Methods.

### Transposon mutagenesis, mutant analysis, and reporter gene fusions

A library of *Pe*299R transposon mutants was generated with the EZTn*5* transposome system (Epicentre, Madison, USA). Individual colonies were screened by low-magnification microscopy for impaired symplasmata formation on M9 glucose agar plates (see Supplementary Methods online for details). For selected mutants, transposon-flanking DNA was sequenced, mapped to the *Pe*299R genome (Genbank accession ANKX00000000), and used for gene complementation assays, as described in Supplementary Methods. For several candidate genes, we constructed transcriptional reporter fusions by cloning DNA fragments encompassing ~100 bp downstream of the start codon as well as the presumed operator/promoter region (300–600 bp upstream of the start codon). We used the following primer pairs for the genes *lrhA*, *rfbX3*, *cspD*, *gshA* and *phzI*, respectively (amplicon size indicated in brackets): lrhA-for2/lrhA-rev2 (670 bp); pst-for2/pst-rev2 (710 bp); cspD-for1/cspD-rev2 (640 bp); yqaB-for1/gshA-rev1 (569 bp); phzR-for2/phzI-rev1 (443 bp) (Supplementary Table S7). Amplicons were digested with restriction enzymes *Eco*RI (or *Sac*I, or *Bam*HI) and *Xho*I and inserted into vector pPROBE’-gfp[tagless] or pPROBE’-gfp[AAV] (Table S5) cut with enzymes *Eco*RI (or *Sac*I, or *Bam*HI) and *Sal*I. The ligation product, which confers resistance to kanamycin, was introduced into *E. coli* TOP10 cells by heat-shock, and the transformants were selected on LB Km agar plates. Plasmids were extracted and transformed into *Pe*299R by electroporation. For reporter analyses, *Pe*299R carrying the empty vector pPROBE’-gfp[tagless] or one of the reporter plasmids (Supplementary Table S6) was grown overnight in 3 ml LB Km. Five ml of fresh LB with antibiotics was inoculated with 1/200 of the o/n culture and incubated at 30 °C with shaking until cultures reached mid-exponential phase (2–3 hours). Cells were harvested at 2,500 g for 10 min, washed twice with M9, and diluted in M9 to an OD_600_ of 0.2. In a 250 ml-glass flask, 20 ml of M9G Km was inoculated with 200 μl of this cell suspension and incubated at 30 °C with shaking. After 0, 90, 180, 240, 360 and 480 min, the flask was sampled to measure GFP fluorescence in individual bacteria using an Accuri C6 flow cytometer (BD Biosciences, San Jose, USA). For this, we defined a gate using the forward and side scattered light data, which gives information about the size and shape of the bacteria, and recorded the intensity of green fluorescence (FL1-A) in 10,000 gated cells per sample. The mean green fluorescence (mean FL1-A) expressed by bacteria was calculated with the CFlow software (BD Biosciences).

### Quantification of symplasmata concentration

From overnight cultures of *Pe299R* and mutants grown in LB medium with appropriate antibiotics, 25 μl was inoculated into 5 ml of fresh LB and incubated for 3 hours at 30 °C with shaking. Cells were harvested by centrifugation at 2,500 g for 10 min, washed twice with M9 devoid of carbon source and resuspended in the same medium to an OD_600_ of 0.2. From this bacterial suspension, 400 μl was added to 20 ml of M9G in a 250 ml-flask, and incubated at 30 °C with shaking. After various periods of incubation, a sample from the culture was mixed with Indian ink (Sanford, Oak Brook, USA) in a 9:1 proportion. Symplasmata were identified as cell clusters with a clear capsule surrounding them and quantified in a hemacytometer (Sigma-Aldrich, St-Louis, USA).

### Chemical treatment of symplasmata

After centrifugation of an overnight culture of *Pe299R* in M9G at 2,500 g for 5 min, the top of the cell pellet (enriched for symplasmata) was collected by pipetting and mixed 1:40 with 50 mM sodium acetate at pH 5.2 in the presence or absence of 10 mM sodium metaperiodate (Sigma-Aldrich). After one hour of incubation at 37 °C, the clusters were analyzed by mixing with Indian ink and microscopy, as described above.

### Electron microscopy

For scanning electron microscopy, 1 mL of an overnight M9G culture of *Pe299R* was harvested by centrifugation at 2,500 g for 5 min. After removal of the supernatant by pipetting, Karnovsky fixative^57^ was added (a volume approximately the size of the pellet) and the sample was incubated overnight at 4 °C. Further sample preparation was done by the Electron Microscopy Laboratory of the Department of Pathology and Laboratory Medicine, School of Medicine, University of California at Davis. In short, the bacterial pellet was rinsed in 0.1 M Sorensen’s buffer prior to application to coverslips coated with polylysine. After one hour of settling, cells were rinsed again with buffer, then dehydrated in increasing concentrations of ethanol. Coverslips were dried in a Tousimis 931 GL critical point dryer (Tousimis Research Corporation, Rockville, USA), mounted on specimen support stubs using carbon tape, and gold-coated using a Pelco SC-7 sputter coater (Ted Pella, Inc., Redding, USA). Samples were examined using a XL30 TMP microscope (Philips, Eindhoven, Netherlands). For transmission electron microscopy, 20 ml of an overnight M9G culture of *Pe299R* was passed three times through the same nylon net filter with an 11 μm pore size (Millipore, Hayward, USA). The filter was transferred to a 15-ml Falcon tube containing 2 ml of PBS, and the bacteria were resuspended by vortexing. This suspension was centrifuged at 2,500 g for 5 min, supernatant was removed, 100 μl of Karnosky fixative was added, and cells were stored at 4 °C until further processing^58^,^59^ by the Electron Microscopy Laboratory. Briefly, the pellet was rinsed in 0.1 M phosphate buffer, then fixed with a solution of 1% osmium in phosphate buffer for one hour. The sample was dehydrated by serial incubation in solutions with increasing concentration of acetone. Resin was infiltrated in the sample and polymerized at 70 °C for 90 min. Ultrathin sections were cut using an ultramicrotome, prepared and analyzed with a CM120 electron microscope (Philips) and a MegaScan 795 camera (Gatan, Pleasanton, USA).

### RNA extractions and RNA-Seq analysis

*Pe299R* wildtype and *lrhA*::Tn5 strains were grown overnight in 5 ml of LB Rif at 30 °C with shaking. Five ml of fresh LB with the same antibiotic were inoculated with 1/200 of preculture and incubated under the same conditions. Bacterial cultures were harvested in mid-log phase after ~3 hours of incubation, by centrifugation at 2,500 g for 10 min. Supernatant was discarded, the pellet rinsed twice in M9 minimal medium devoid of carbon source, resuspended in the same medium and diluted to an OD_600_ of 0.2. Twenty ml of M9GCA in a 250-ml glass flask was inoculated with 100 μl of bacterial suspension and incubated as previously. Once OD_600_ reached ~0.5 (~8 hours of incubation), cultures were passed three times through the same nylon net filter with an 11 μm pore size (Millipore). A 2-ml filtrate sample (corresponding to ~5 × 10^8^ bacteria) was mixed well with 4 ml of RNA Protect solution (Qiagen) and kept at room temperature until further use, but for no longer than 30 min. In parallel, four 40-ml aliquots of M9G in 500-ml glass flasks were inoculated with 200 μl and incubated under the same conditions. After ~13–15 hours of incubation, cultures were pooled together as OD_600_ was ~0.5. The pooled suspension was filtered as describe above, the filtrate was discarded, and three times 20 ml of M9 were passed through the filter to wash the cells on the filter. The filter was transferred into a 15-ml Falcon tube, to which 2 ml of M9 (no carbon source) and 4 ml of RNA Protect was added prior to vortexing. The tube was placed in a rack in an ultrasonic cleaner (Model 5510, Branson Ultrasonics Corporation, Danbury, CT, USA) for 5 min. Two ml of filtrate were treated as described previously. Samples of cells mixed with RNA Protect were centrifuged at 5,000 g for 10 min, supernatant was discarded, then 200 μl of RNAse-free TE buffer (USB, Cleveland, OH, USA) containing 1 mg of lysozyme per ml was added to resuspend the cells. Samples were incubated at room temperature for 10 min with 10 sec of vortexing every 2 min. Total RNA was further extracted using a RNEasy minikit (Qiagen), following the manufacturer’s instructions. Total RNA was finally eluted in 50 μl of RNAse-free water and quantified by NanoDrop (Thermo Fisher Scientific, Waltham, MA, USA). Quality control, rRNA depletion, cDNA library preparation, DNA sequencing, and quantitative transcript analysis were performed using established protocols, as described in Supplementary Methods.

## Additional Information

**How to cite this article**: Tecon, R. and Leveau, J. H. J. Symplasmata are a clonal, conditional, and reversible type of bacterial multicellularity. *Sci. Rep.* **6**, 31914; doi: 10.1038/srep31914 (2016).

## Supplementary Material

Supplementary Information

Supplementary Table S3

Supplementary Table S4

Supplementary Movie S1
