# Supplementary material for: Symplasmata are a clonal, conditional, and reversible type of bacterial multicellularity
Source: Sci Rep. 2016 Aug 18;6:31914. doi: 10.1038/srep31914 (PMC4989142; doi:10.1038/srep31914)
Supplement: Supplementary Information [file srep31914-s1.doc]

**Title:** Symplasmata are a clonal, conditional and reversible type of bacterial multicellularity

**Authors:** Robin Tecon, Johan Leveau

**Supplementary Information**

**Supplementary Methods** *pp.2-9*

**Suppl. Fig. S1.** Lipophilic staining of *Pe*299R cells. *p.10*

**Suppl. Fig. S2.** Metabolic cell activity inside symplasmata. *p.11*

**Suppl. Fig. S3.** Plasmid complementation of *Pe*299R transposon mutants. *p.12*

**Suppl. Fig. S4.** Swimming motility of *Pe*299R and transposon mutants. *p.13*

**Suppl. Fig. S5.** Various stages of symplasmata formation in *Pe*299R. *p.14*

**Suppl. Fig. S6.** Hierarchical clustering of RNA-Seq data. *p.15*

**Suppl. Fig. S7.** Attachment of symplasmata to a gel surface. *p.16*

**Suppl. Movie S1 legend.** *Pe*299R bacteria thrown out of a symplasmatum. *p.17*

**Suppl. Table S1.** Carbohydrate composition of extracellular polymeric substance (EPS) associated with symplasmata of *Pe*299R obtained by GC/MS after acid methanolysis. *p.18*

**Suppl. Table S2.** Transposon mutants of *Pe*299R showing abolished or impaired clustering phenotype. *p.19*

**Suppl. Table S3 legend**. Differentially expressed genes in *Pe*299R (single cells + symplasmata) and *lrhA*::Tn*5* grown in M9G. *p.20*

**Suppl. Table S4 legend**. Differentially expressed genes between 299R individual cells and 299R cells in symplasmata, from the same culture in M9G. *p.21*

**Suppl. Table S5.** Symplasmata-forming bacterial isolates from the lettuce phyllosphere. *p.22*

**Suppl. Table S6.** Bacterial strains and plasmids used in this study. *p.23*

**Suppl. Table S7**. List of primers used in this study. *p.24*

**Supplementary Methods**

*Symplasmata formation by fluorescently labeled* P. eucalypti

Strains *Pe*299R (pFRU48) and *Pe*299R (pFRU97) constitutively express the green and the red fluorescent protein, respectively . They were grown separately from plate in LB Km overnight, diluted 200 times in fresh LB Km and incubated for 4 hours at 30˚C with shaking. The two cultures were centrifuged at 2,500 g for 10 min, washed twice with M9 devoid of carbon source and resuspended in M9 to an optical density at 600 nm (OD_600_) of 0.2. We mixed equal volumes of the two cell suspensions, and inoculated 400 μl of this mixture into 20 ml of M9 containing 0.4% glucose (M9G) and 50 μg of kanamycin per ml suspension. After 18 hours of incubation at 30˚C with shaking, a sample of the bacterial culture was observed with an Axio Imager.M2 epifluorescent microscope (Zeiss, Oberkochen, Germany), using a 20x objective (EC Plan-NEOFLUAR 20x/0.5, Zeiss) and a 63x objective (EC Plan-NEOFLUAR 63x/1.25 Oil, Zeiss). We took fluorescent and phase contrast images sequentially with an AxioCam MRm monochrome camera (Zeiss), using a rhodamine filter cube (exciter: 546/12; emitter: 607/80; beamsplitter 560) and a GFP filter cube (exciter: 470/40; emitter: 525/50; beamsplitter 495) to visualize red and green fluorescence, respectively.

*Membrane staining*

The lipophilic styryl dye FM®1-43 (Molecular Probes, Eugene, OR, USA) was added (1 mM final concentration) to a suspension of *Pe299R* bacteria in M9. Ten μl of this suspension was inoculated onto a 1 cm^2^ gel patch of M9G 1% agarose MP (Roche Diagnostics, Indianapolis, USA), then incubated in the dark at room temperature for 24 hours. The bacteria were observed with an Axio Imager.M2 epifluorescent microscope and a 63X objective. FM®1-43 fluorescence was visualized using the GFP filter cube.

*EPS analysis*

Four 50-ml cultures of *Pe*299R were grown overnight in M9G, transferred to 50-ml Falcon tubes and centrifuged at 2,500 g for 10 min. The supernatant (containing the symplasmata) was decanted into new centrifuge tubes and centrifuged at max speed (7,197 g) for 10 min. The supernatant was decanted, and the resuspended pellets were transferred to 2-ml microcentrifuge tubes and centrifuged at full speed (19,090 g) for 2 min. Excess supernatant was removed by pipetting. The pellets were composed of single cells and symplasmata, visible as two distinct fractions. The symplasmata fraction (on top) was pipetted to 1.5 ml-microcentrifuge tubes, instant-frozen in liquid nitrogen, and stored at -20˚C. Analysis of the Extracellular Polymeric Subsance (EPS) was conducted by the Complex Carbohydrate Research Center, University of Georgia. The four symplasmata samples were pooled for the analysis and compared to a non-symplasmata control, which was *Pe*299R cells grown in LB. Results of the analysis are shown in Table S1.

*Swimming motility assay*

We inoculated ‘motility’ plates (per liter: 10 g tryptone; 5 g NaCl; 2.5 g agar) by pipetting 10 μl of an overnight LB culture of *Pe*299R at the center of the plate. The plates were sealed with parafilm and incubated at 28˚C for 5 hours. We measured the diameter of the dispersion halo with a ruler at the end of the incubation period. We calculated a mean diameter from two plates per strain in each independent experiment.

*Transposon mutagenesis*

In a 250 ml flask, 70 ml of LB were inoculated with 1% (700 μl) of an overnight preculture of *Pe*299R, and incubated at 30˚C with shaking until OD_600_ reached 0.5-0.6 (~3 hours). Two times 30 ml of culture were transferred to 50-ml centrifuge tubes (Fisher Scientific, Pittsburgh, USA) and incubated on ice for 30 min. The cells were then harvested by centrifugation at 2,500 g for 15 min at 4˚C. Supernatant was removed and cells were resuspended in 2 x 25 ml of ice cold Milli-Q water and centrifuged as before. We repeated the operation to resuspend the cells in 2x 10 ml of ice-cold MQ water, then in 2 x 5 ml of ice-cold 10% glycerol in MQ water. After the final centrifugation, the cells were resuspended in ~200 μl of 10% glycerol (OD_600_=~30). Fifty-μl aliquots of this suspension we redispensed in microcentrifuge tubes and mixed with no more than 1 μl of EZTn5 transposome solution (Epicentre, Madison, USA). After a few minutes on ice, the solution was transferred to sterile 0.1 cm-electroporation cuvettes (BioRad, Hercules, USA) and incubated on ice for 2-3 min. Electroporation was performed with a BioRad Gene Pulser apparatus with the following settings: 25 μF, 200 ohms, and 1.8 kV. Immediately after electroporation, 1 ml of SOC medium was added to the cuvette and gently mixed by pipetting. The suspension was transferred to a 17 mm x 100 mm polypropylene tube (Fischer Scientific) and incubated for 1 hour at 30˚C with gentle shaking. The suspension or dilutions thereof were plated on LB agar plates with 50 μg of kanamycin per ml and incubated overnight for the selection of transposon mutants. Three independent rounds of EZTn5 electroporations were performed, each yielding thousands of transposon mutants per μl of EZTn5 transposome. Transposon mutants were transferred from LB agar plates to M9G plates with 1% agarose MP (Roche Diagnostics, Indianapolis, USA). In total, 4981 mutants were screened for the absence of symplasmata in bacterial colonies. Screening was done with a 10x objective (EC Plan-NEOFLUAR 10x/0.25, Zeiss). Colonies in which no clusters were observed were tested again on M9G 1% agarose plate and in M9G liquid culture for confirmation of the phenotype.

*Identification of transposon insertions and genetic complementations*

Genomic DNA was isolated from 1-ml of overnight cultures of selected transposon mutants (Table S2) using the kit DNeasy (Qiagen, Maryland, USA) and resuspended in 100 μl of elution buffer. Three μl of DNA (~200-600 ng) in a total volume of 10 μl was further digested with 10 U of restriction enzyme (*Pst*I or *Sal*I) for 1 hour at 37˚C, followed by thermal inactivation at 80˚C for 20 min. One μl of digested DNA was self-ligated in a final volume of 10 μl with 1,000 U of T4 DNA ligase, for 2 hours at room temperature. Two μl of ligated DNA in a final volume of 20 μl was amplified by PCR using primers KAN-2 FP1 and KAN-2 RP1 (Table S7) at a concentration of 500 nM. The reaction cycle was as follows: 95˚C for 3 min; 30x [95˚C for 30 sec, 55˚C for 30 sec, 72˚C for 90 sec]; 72˚C for 3 min. Amplicons were visualized on a 0.8 % agarose gel, and purified from the gel using the Qiaquick kit (Qiagen). PCR products were sequenced using primers KAN-2 FP1 and KAN-2 RP1. The DNA sequence was blasted against the NCBI database and against the genome of *Pe*299R (GenBank Assembly ID: GCA_000330765.1).

Complementation of transposon mutants was performed by cloning amplified full target gene(s) and cognate operator/promoter region into cloning vector pME6031 (Table S6). We used the following primer pairs (Table S7) to amplify *lrhA*, *rfbX3*, *cspD* and *phzI,* respectively: lrhA-for2/lrhA-rev1; pst-for2/pst-rev1; pst-for2/pst-rev3; cspD-for1/cspD-rev1; phzR-for2/phzI-rev2. In a final volume of 20 μl, approximately 30 ng of *Pe*299R genomic DNA was PCR-amplified with 500 nM of each primer and the following cycle: 95˚C for 3 min; 30x [95˚C for 30 sec, 55˚C for 30 sec, 72˚C for 45 to 90 sec]; 72˚C for 5 min. PCR products were visualized on 0.8% agarose gel and purified with Qiaquick extraction kit (Qiagen) in 50 μl of elution buffer. PCR products were digested with appropriate restriction enzymes and ligated into pME6031 that was digested with the same enzymes and dephosphorylated with 1 Unit of shrimp alkaline phosphatase for 45 min at 37˚C. *E. coli* TOP10 cells were transformed with the ligation reaction using heat-shock , and transformants were selected on LB agar plates with 10 μg of tetracycline per ml. Transformants were grown in LB liquid cultures with tetracycline, and plasmid DNA was isolated using GeneJet (Fischer Scientific). Isolated plasmids were digested and checked on gel for the presence of the correct insert size. Electrocompetent cells of 299R and mutant strains were prepared as described previously, and transformed with ~50 ng of plasmid. Transformants were selected on LB agar plates with tetracycline and the presence of the plasmid was checked as described above.

*RNA extractions for transcriptomic studies*

*Pe*299R wildtype and *lrhA*::Tn*5* strains were grown overnight in 5 ml of LB with 20 μg of rifampycin per ml at 30˚C with shaking. Five ml of fresh LB with the same antibiotic were inoculated with 1/200 of preculture and incubated under the same conditions. Bacterial cultures were harvested in mid-log phase after ~3 hours of incubation, by centrifugation at 2,500 g for 10 min. Supernatant was discarded, the pellet was rinsed twice in M9 minimal medium devoid of carbon source, resuspended in the same medium and diluted to an OD_600_ of 0.2. Twenty ml of M9GCA in a 250-ml glass flask was inoculated with 1/200 (100 μl) of bacterial suspension and incubated as previously. Once OD_600_ reached ~0.5 (~8 hours of incubation), cultures were passed three times through the same nylon net filter with an 11 μm pore size (Millipore, Hayward, USA). A 2 ml filtrate sample (corresponding to ~5 x 10^8^ bacteria) was mixed well with 4 ml of RNA Protect solution (Qiagen) and kept at room temperature until further use, but for no longer than 30 min. In parallel, four times 40 ml of M9G in 500-ml glass flasks were inoculated with 1/200 (200 μl) and incubated under the same conditions (the total volume was higher than previously in order to provide enough material for RNA extraction from clusters). After ~13-15 hours of incubation, cultures were pooled together as OD_600_ was ~0.5. The pooled suspension was filtered as describe above, the filtrate was discarded, and three times 20 ml of M9 were passed through the filter to wash the cells on the filter. The filter was transferred into a 15-ml Falcon tube, to which 2 ml of M9 (no carbon source) and 4 ml of RNA Protect was added prior to vortexing. The tube was placed in a rack and incubated in an ultrasonic cleaner (Model 5510, Branson Ultrasonics Corporation, Danbury, CT, USA) for 5 min. Two ml of filtrate was treated as described previously. Samples of cells mixed with RNA Protect were centrifuged at 5,000 g for 10 min, supernatant was discarded, then 200 μl of RNAse-free TE buffer (USB, Cleveland, OH, USA) containing 1 mg of lysozyme per ml was added to resuspend the cells. Samples were incubated at room temperature for 10 min with 10 sec of vortexing every 2 min. Total RNA was further extracted using a RNEasy minikit (Qiagen), following the manufacturer’s instructions. Total RNA was finally eluted in 50 μl of RNAse-free water and quantified by NanoDrop (Thermo Fisher Scientific, Waltham, MA, USA).

*Construction of cDNA libraries, sequencing and RNA-Seq analysis*

The DNA Technologies Core Facility at UC Davis performed a quality check (RNA Integrity Number) of the total RNA samples using an Agilent 2100 Bioanalyzer and further constructed the libraries. For each library, 1.5 μg of total RNA was treated with the Ribo-Zero rRNA removal kit for bacteria (Epicentre) following the manufacturer’s instructions. After rRNA depletion, libraries were prepared with the Illumina mRNA Sample Prep Reagent kit (Illumina, San Diego, USA) following the manufacturer’s instructions. Briefly, cDNA was synthesized from fragmented RNA, then end-repaired and phosphorylated. Blunt-end fragments were A-tailed and sequencing adapters were ligated to the fragments. Library fragments were enriched with 12 cycles of PCR. Libraries were pooled and sequenced on a HiSeq2000 platform, and single reads of 50 cycles were collected. FASTQ files were generated and demultiplexed using the Illumina pipeline (CASSAVA 1.8).

RNA reads were aligned in the web-based platform Galaxy (www.galaxyproject.org) to the draft genome sequence of *Pe*299R using BWA (version 1.2.3, with default settings. The alignments were analyzed using the R-based Bioconductor software ([www.bioconductor.org](http://www.bioconductor.org/)). Reads were imported into R and counted using the package GenomicRanges and the function summarizeOverlaps (union method). Data was exported to an Excel worksheet, and the individual gene counts were further standardized by the total number of counted reads in each library and by the gene length in order to obtain RPKM values. The raw counts from different replicates were combined in a table and the statistics for differentially expressed genes were calculated using the DESeq package .

**References**

Anders, S., and Huber, W. Differential expression analysis for sequence count data. *Genome Biology* **11**: R106 (2010).

Aziz, R., *et al*. The RAST Server: Rapid Annotations using Subsystems Technology. *BMC Genomics* **9**: 75 (2008).

Brandl, M., and Lindow, S. Cloning and characterization of a locus encoding an indolepyruvate decarboxylase involved in indole-3-acetic acid synthesis in *Erwinia herbicola*. *Appl. Environ. Microbiol*. **62**: 4121-4128 (1996).

Heeb, S., *et al.* Small, stable shuttle vectors based on the minimal pVS1 replicon for use in Gram-negative, plant-associated bacteria. *Mol. Plant-Microbe Interact.* **13**: 232-237 (2000).

Lawrence, M., *et al.* Software for computing and annotating genomic ranges. *PLoS Comput. Biol*. **9**: e1003118 (2013).

Leveau, J.H.J., and Lindow, S.E. Predictive and interpretive simulation of green fluorescent protein expression in reporter bacteria. *J. Bact.* **183**: 6752-6762 (2001).

Li, H., and Durbin, R. Fast and accurate short read alignment with Burrows–Wheeler transform. *Bioinformatics* **25**: 1754-1760 (2009).

Miller, W.G., Leveau, J.H.J., and Lindow, S.E. Improved gfp and inaZ broad-host-range promoter-probe vectors. *Mol. Plant-Microbe Interact.* **13**: 1243-1250 (2000).

Remus-Emsermann, M.N.P., Kim, E.B., Marco, M.L., Tecon, R., and Leveau, J.H.J. Draft genome sequence of the phyllosphere model bacterium *Pantoea agglomerans* 299R. *Genome Announcements* **1**(1) (2013).

Sambrook, J., and Russel, D.W. *Molecular Cloning, A Laboratory Manual* (Cold Spring harbor, 2001).

Tecon, R., and Leveau, J.H.J. The mechanics of bacterial cluster formation on plant leaf surfaces as revealed by bioreporter technology. *Environ. Microbiol.* **14**: 1325-1332 (2012).


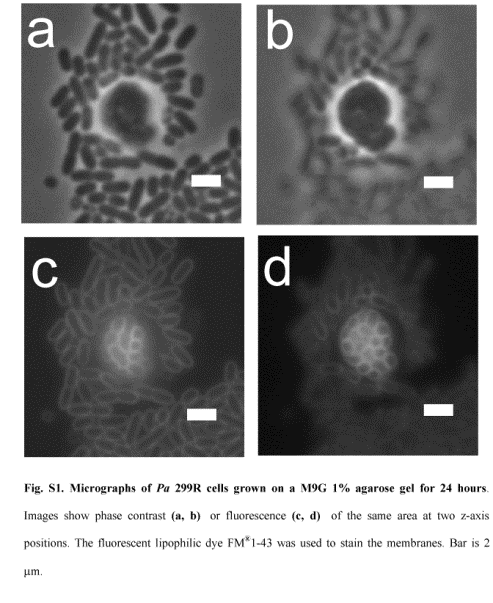


**Fig. S1.** **Lipophilic staining of** ***Pe*299R cells.** Bacteria were grown on a M9G agarose 1% gel for 24 hours to stimulate symplasmata formation. The fluorescent lipophilic dye FM®1-43 was used to stain the cell membranes. Micrographs show phase contrast (**a**, **b**) or fluorescence signal (**c**, **d**) from the same area at two z-axis positions. The layers surrounding the symplasmatum are not stained by FM®1-43. Bar is 2 μm.


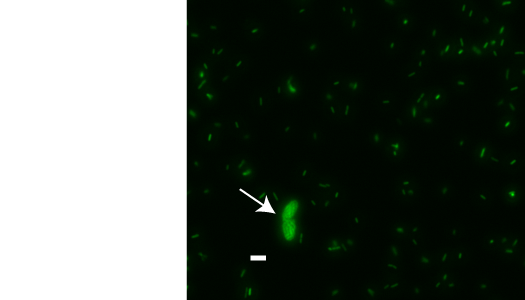


**Fig. S2.** **Metabolic cell activity inside symplasmata.** We used the reporter strain *Pe*299R::JBA28 (pCPP39) to assess bacterial metabolic activity. In this system, GFP expression can be stimulated by exposure to IPTG. Bacteria were grown in liquid M9G cultures overnight in order to stimulate symplasmata formation. Under those conditions no GFP fluorescence was produced. We then added 1 mM IPTG to the medium and incubated the cells for 1 hour. Micrograph shows *Pe*299R::JBA28 (pCPP39) reporter cells expressing GFP after IPTG induction. GFP fluorescence, shown with pseudo green color, was detectable both in single cells and in cells within a symplasmatum (white arrow). Bar is 5 μm.


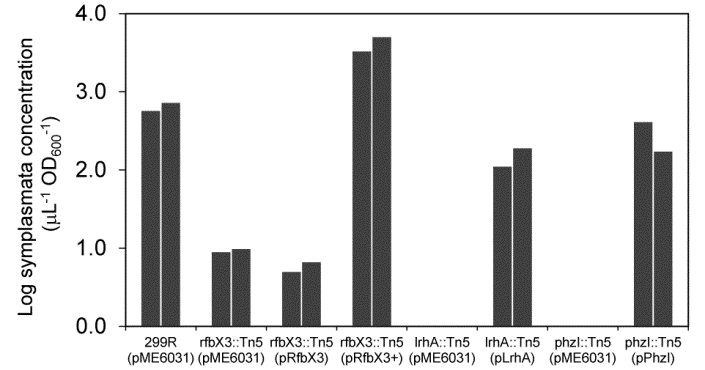


**Fig. S3. Plasmid complementation of *Pe*299R transposon mutants**. Bacteria were transformed with an empty vector (pME6031) or with the same vector containing the cognate wildtype gene under the control of its original promoter (see Table S6). Vector pRfbX3+ contains both the gene *rfbX3* and its immediate downstream gene. Symplasmata formation was induced by overnight growth in liquid M9G, and results from duplicate experiments are shown. Tetracycline at a concentration of 10 μg/ml was used to ensure plasmid maintenance. We noted, however, that the addition of tetracycline slightly decreased the total number of symplasmata observed in the wild-type strain.


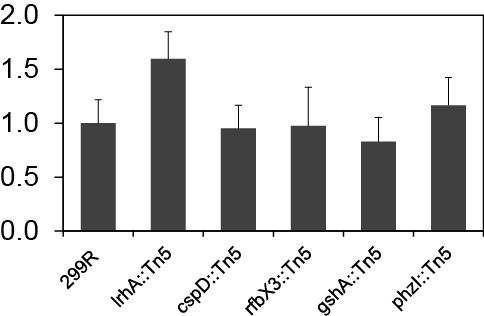


**Fig. S4. Swimming motility of *Pe*299R and transposon mutants**. Ten μl of overnight preculture was pipetted on the center of a motility plate, and we measured the diameter of the dispersion halo after 5 hours of incubation. Values were normalized to the average diameter reached by 299R. Error bars indicate standard deviations calculated from at least 5 independent experiments.


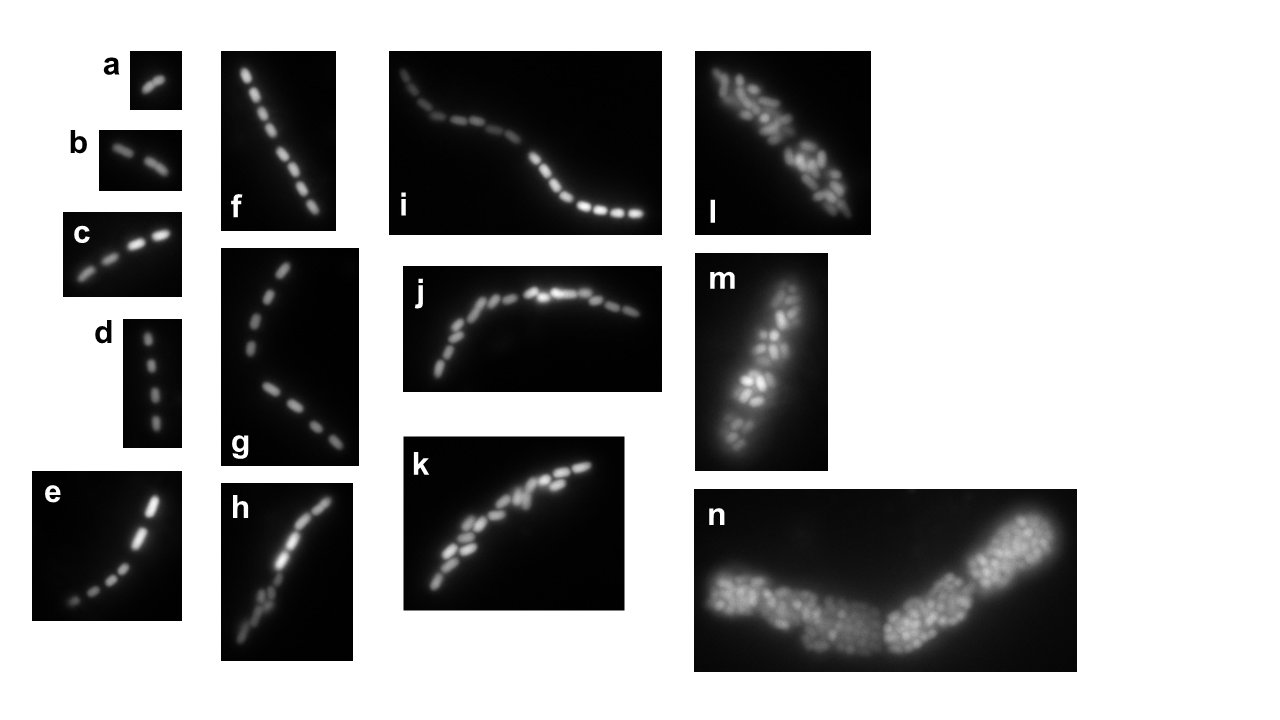


**Fig. S5. Various stages of symplasmata formation in *Pe*299R**. Bacteria were grown in a M9G shaking culture and carried a fusion of the symplasmata-specific *rfbX3* promoter to the *gfp* reporter gene. Micrographs show clusters of 1 (**a**), 2 (**b**), 4 (**c-d**), 8 (**f-h**), 16 (**i-k**) or more (**l-n**) cells, including intermediate clusters e.g. 4+2 (**e**) or 6+4 (**h**) cells, linearly aligned in small clusters (**a-j**), more randomly organized in large clusters (**k-n**).


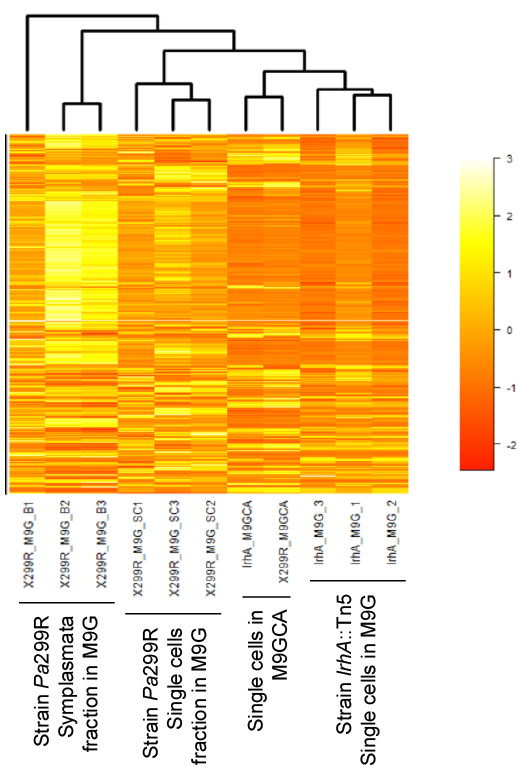


**Fig. S6. Hierarchical clustering of RNA-Seq data.** Three biological replicates were used per condition in M9G cultures, and one for cultures in M9GCA. Data (RPKM values for all genes) were clustered and heatmap (intensity plot) was produced using the Heatplus package in R, with the method average linkage (UPGMA).


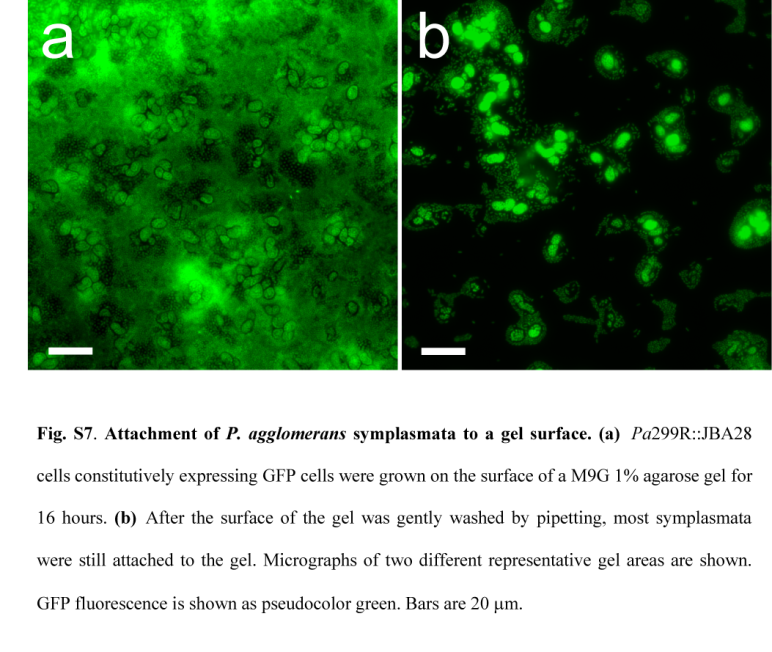


**Fig. S7**. **Attachment of symplasmata to a gel surface.** (**a**) *Pe*299R::JBA28 cells constitutively expressing GFP were grown on the surface of a M9G 1% agarose gel for 16 hours. (**b**) After the surface of the gel was gently washed by pipetting, most symplasmata were still attached to the gel. Micrographs of two different representative gel areas are shown. GFP fluorescence is shown as pseudo-color green. Bar is 20 μm.

**Movie S1. *Pe*299R bacteria thrown out of a symplasmatum.** Symplasmata grown in M9G batch cultures were first isolated by filtration, then transferred to the surface of a M9GCA 1% agarose gel and incubated at room temperature. The movie shows growth of bacteria outside of the symplasmatum starting 3 hours post-transfer and ending 4 hours later.

*Movie provided as a mov file.*

**Table S1.** **Carbohydrate composition of extracellular polymeric substance (EPS) associated with symplasmata of *Pe*299R obtained by GC/MS after acid methanolysis.** Samples were analyzed by the Center for Plant and Microbial Complex Carbohydrates which is supported by funds from the Department of Energy (DE-FG02-93ER-20097).

|  | **Sample^a^** |  | **Fraction^b^** |  | **Glycosyl residues** |  | **Mol %^c^** |
| --- | --- | --- | --- | --- | --- | --- | --- |
|  | Symplasmata  Supernatant 1  Supernatant 2  Supernatant 3 |  | HMW*  LMW  HMW*  LMW  HMW - peak 1*  LMW – peak 2  LMW – peak 3 |  | Rhamnose  Glucuronic Acid  Galactose  Glucose  KDO  Heptose  Xylose  Glucose  Rhamnose  Xylose  Galacturonic Acid  Mannose  Galactose  Glucose  Heptose  Xylose  Mannose  Xylose  Mannose  Glucose  Glucose  Inositol  Xylose  Glucose  Inositol |  | 56.0  2.2  12.1  21.4  3.1  5.3  8.2  91.8  45.8  19.6  5.5  2.5  1.9  15.5  9.3  22.4  77.6  20.5  31.2  48.3  57.5  42.5  24.0  22.4  53.6 |
|  | Single cells  Supernatant 1  Supernatant 2 |  | HMW  LMW  HMW |  | Rhamnose  Glucuronic Acid  Galactose  Glucose  Heptose  Glucose  Rhamnose |  | 6.1  8.2  34.9  45.2  5.6  100.0  100.0 |

^a^ Supernatants were obtained sequentially from a single pooled sample enriched for either symplasmata or single cells of *P. eucalypti*.

^b^ HMW: high molecular weight, ~190-300KDa ; LMW: low molecular weight, <10 kDa.

^c^ Values are expressed as mole percent of total carbohydrates. The total percentage may not add to exactly 100 % due to rounding.

* Glycerol detected in the sample.

**Table S2.** **Transposon mutants of *Pe*299R showing abolished or impaired clustering phenotype.**

| **Gene insertion** | **NCBI Gene  locus tag** | **Predicted protein** | **NCBI Protein accession nr.** | **Gene size [bp]** | **Phenotype** | **Nb of mutants** |
| --- | --- | --- | --- | --- | --- | --- |
| *lrhA* | [F385_RS17575](http://www.ncbi.nlm.nih.gov/nuccore/440760383) | LysR-type transcriptional regulator | ELP23565 | 924 | Total absence of symplasmata formation | 3 |
| *rfbX3* | [F385_RS21230](http://www.ncbi.nlm.nih.gov/nuccore/440761114) | Polysaccharides biosynthesis and transport protein | ELP22860 | 1257 | Reduced rate of symplasmata formation | 2 |
| *gshA* | [F385_RS02625](http://www.ncbi.nlm.nih.gov/nuccore/440757482) | Glutamate cysteine ligase | ELP26396 | 1608 | Total absence of symplasmata formation | 1 |
| *phzI* | [F385_RS04930](http://www.ncbi.nlm.nih.gov/nuccore/440757804) | N-acyl-L-homoserine lactone synthetase | ELP26088 | 612 | Total absence of symplasmata formation | 1 |
| *cspD* | [F385_RS03285](http://www.ncbi.nlm.nih.gov/nuccore/440757605) | Cold shock protein | ELP26278 | 225 | Reduced rate of symplasmata formation | 1 |
| *yhdP* | [F385_RS20315](http://www.ncbi.nlm.nih.gov/nuccore/440760990) | Putative exported protein | ELP22941 | 3840 | Reduced rate of symplasmata formation | 1 |
| *rodA* | [F385_RS16900](http://www.ncbi.nlm.nih.gov/nuccore/440760304) | Rod-shape determining protein | ELP23628 | 1119 | Reduced rate of symplasmata formation, abnormal cell wall morphology | 1 |

**Table S3**. **Differentially expressed genes in *Pe*299R (single cells + symplasmata) and *lrhA*::Tn5 grown in M9G, as analyzed by RNA-Seq**. Genes that are thought to belong to the same transcriptional unit are grouped together in shaded boxes, with the first transcribed gene on the first line. Positive fold-change indicates that the gene is upregulated in the *lrhA* mutant.

*Table provided as an Excel file.*

**Table S4**. **Differentially expressed genes between 299R individual cells and 299R cells in symplasmata, from the same culture in M9G.** Genes that are thought to belong to the same transcriptional unit are grouped together in shaded boxes, with the first transcribed gene on the first line. Positive fold-change indicates that the gene is upregulated in symplasmata.

*Table provided as an Excel file.*

**Table S5**. **Symplasmata-forming bacterial isolates from the lettuce phyllosphere**

| **Isolates** | **Classification^1^** |
| --- | --- |
| 1A2,1A4,1B3,1B4,1C2,1C4,1D3,1G3 | *Pantoea agglomerans* |
| 1B8,1C5,1C7,1D5,1,D8,1F3 | *Pantoea anthophila* |

^1^Classification based on Seqmatch analysis (https://rdp.cme.msu.edu/) using high-quality 16S rRNA gene sequences that were obtained using primer 799f as a sequencing primer on pA-1492r PCR amplicons from individual isolates.

**Table S6.** **Bacterial strains and plasmids used in this study.**

|  | **Strain or plasmid** |  | **Description^a^** |  | **References** |  |
| --- | --- | --- | --- | --- | --- | --- |
|  | *P. eucalypti* |  |  |  |  |  |
|  | 299R |  | Rif^R^ |  |  |  |
|  | 299R::JBA28 |  | Constitutive expression of GFP from a chromosomal insertion, Rif^R^, Km^R^ |  |  |  |
|  | 299R::JBA28 (pCPP39) |  | IPTG-inducible expression of GFP, Rif^R^, Km^R^, Tet^R^ |  |  |  |
|  | *lrhA*::Tn5 |  | 299R::EZTn5; Rif^R^, Km^R^ |  | This study |  |
|  | *rfbX3*::Tn5 |  | 299R::EZTn5; Rif^R^, Km^R^ |  | This study |  |
|  | *cspD*::Tn5 |  | 299R::EZTn5; Rif^R^, Km^R^ |  | This study |  |
|  | *gshA*::Tn5 |  | 299R::EZTn5; Rif^R^, Km^R^ |  | This study |  |
|  | *phzI*::Tn5 |  | 299R::EZTn5; Rif^R^, Km^R^ |  | This study |  |
|  |  |  |  |  |  |  |
|  | pFRU48 |  | GFP-tagging vector, Km^R^ |  |  |  |
|  | pFRU97 |  | DsRed-tagging vector, Km^R^ |  |  |  |
|  | pME6031 |  | Cloning vector developed for *Pseudomonas* spp., Tet^R^ |  |  |  |
|  | pRfbX3 |  | pME6031 carrying a full copy of the *rfbX3* gene under the control of its cognate promoter. |  | This study |  |
|  | pRfbX3+ |  | pME6031 carrying a full copy of *rfbX3* and its immediately downstream gene under the control of their cognate promoter. |  | This study |  |
|  | pLrhA |  | pME6031 carrying a full copy of the *lrhA* gene under the control of its cognate promoter. |  | This study |  |
|  | pPhzI |  | pME6031 carrying a full copy of the *phzI* gene under the control of its cognate promoter. |  | This study |  |
|  | pPROBE’-gfp[tagless] |  | Broad-host-range promoter-probe vector encoding a stable gfp, Km^R^ |  |  |  |
|  | pPROBE’-gfp[AAV] |  | Broad-host-range promoter-probe vector encoding an unstable gfp, Km^R^ |  |  |  |
|  | pRfbX3-gfp |  | pPROBE’ carrying the rfbX3-gfp transcriptional fusion |  | This study |  |
|  | pLrhA-gfp |  | pPROBE’ carrying the lrhA-gfp transcriptional fusion |  | This study |  |
|  | pGshA-gfp |  | pPROBE’ carrying the gshA-gfp transcriptional fusion |  | This study |  |
|  | pPhzI-gfp |  | pPROBE’ carrying the phzI-gfp transcriptional fusion |  | This study |  |
|  | pCspD-gfp |  | pPROBE’ carrying the cspD-gfp transcriptional fusion |  | This study |  |

^a^ Rif^R^: rifampicin resistance; Km^R^: kanamycin resistance; Tet^R^: tetracycline resistance.

**Table S7**. **List of primers used in this study.**

|  | **Primers** |  | **Primers sequence^a^** |  | **References** |  |
| --- | --- | --- | --- | --- | --- | --- |
|  | KAN-2 FP1 |  | ACCTACAACAAAGCTCTCATCAACC |  | Epicentre |  |
|  | KAN-2 RP1 |  | GCAATGTAACATCAGAGATTTTGAG |  | Epicentre |  |
|  | lrhA-for2 |  | AAAGAATTCGTTTAAGGCCTTGTGATCGC |  | This study |  |
|  | lrhA-for3 |  | AAAGAATTCtcttcaaggcgcttagcctc |  | This study |  |
|  | lrhA-rev1 |  | AAACTCGAGTTACTCTTCGTCATCCAAC |  | This study |  |
|  | lrhA-rev2 |  | AAACTCGAGTTCTGCAGACCGCTGCAGCG |  | This study |  |
|  | pst-for1 |  | AAAGAATTCGGCAGTATTAAAGTGCGCGA |  | This study |  |
|  | pst-rev1 |  | AAACTCGAGCTTGAGCATATCTGACCTCA |  | This study |  |
|  | pst-for2 |  | AAAGAGCTCCTCTCTGACTTTCACGCCCG |  | This study |  |
|  | pst-rev2 |  | AAACTCGAGGGCCAAAAGACTTCGCCACG |  | This study |  |
|  | pst-rev3 |  | AAACTCGAGTATATTGCTGTGTGTGCTTA |  | This study |  |
|  | cspD-for1 |  | AAAGAATTCCACAGATCGCCTTGCCGTGA |  | This study |  |
|  | cspD-rev1 |  | AAACTCGAGGTGGGTTCAGTATCAGGCCA |  | This study |  |
|  | cspD-rev2 |  | AAACTCGAGTGAATGGTGGAGTAGTGCGC |  | This study |  |
|  | yqaB-for1 |  | AAAGGATCCcaggcatggcagtggtggat |  | This study |  |
|  | gshA-rev1 |  | AAACTCGAGCAGCCAGGATAGCGCTTTAG |  | This study |  |
|  | phzR-for2 |  | AAAGAATTCAGAGTTTAGTCAGCGTGAGC |  | This study |  |
|  | phzI-rev1 |  | AAACTCGAGTGTCCTGGCGTACTCAGACG |  | This study |  |
|  | phzI-rev2 |  | AAACTCGAGAGTGGCGGGGGCATATACGC |  | This study |  |
| ^a^ Restriction sites are underlined. *Eco*RI (GAATTC); *Xho*I (CTCGAG); *Sac*I (GAGCTC); *Bam*HI (GGATCC). | | | | | | |
